# Clinical implications of different types of dementia in patients with atrial fibrillation: Insights from a global federated health network analysis

**DOI:** 10.1002/clc.24006

**Published:** 2023-04-10

**Authors:** Riccardo Proietti, José Miguel Rivera‐Caravaca, Raquel López‐Gálvez, Stephanie L. Harrison, Benjamin J. R. Buckley, Francisco Marín, Paula Underhill, Eduard Shantsila, Alena Shantsila, Rhys Davies, Deirdre A. Lane, Gregory Y. H. Lip

**Affiliations:** ^1^ Liverpool Centre for Cardiovascular Science at University of Liverpool, Liverpool Heart & Chest Hospital Liverpool John Moores University Liverpool UK; ^2^ Faculty of Nursing University of Murcia Murcia Spain; ^3^ Department of Cardiology, Hospital Clínico Universitario Virgen de la Arrixaca, Instituto Murciano de Investigación Biosanitaria (IMIB‐Arrixaca), CIBERCV University of Murcia Murcia Spain; ^4^ Department of Cardiovascular and Metabolic Medicine, Institute of Life Course and Medical Sciences University of Liverpool Liverpool UK; ^5^ TriNetX LLC London UK; ^6^ The Walton Centre NHS Foundation Trust, Lower Lane, UK Liverpool

**Keywords:** Alzheimer's disease, atrial fibrillation, bleeding, dementia, stroke

## Abstract

**Background:**

Atrial fibrillation (AF) associates with higher Alzheimer's disease (AD) and vascular dementia risks but the clinical implications have been scarcely investigated. We examined the association between AD or vascular dementia and adverse outcomes in AF patients.

**Methods:**

Cohort study between January 2000 and 2017. AF patients were divided into two groups according to vascular dementia or AD, and balanced using propensity score matching (PSM). During 4‐years of follow‐up, incident intracranial hemorrhages (ICH), the composite of ischemic stroke/transient ischemic attack (TIA), hospitalizations, and all‐cause deaths, were recorded.

**Results:**

Two thousand three hundred seventy‐seven AF patients with dementia (1225 with vascular dementia, and 1152 with AD) were identified. Following a PSM, 615 patients were included in each cohort (i.e., 1:1) and all variables were well‐matched. After PSM, 22 (3.6%) patients with vascular dementia and 55 (8.1%) patients with AD had incident ICH during follow‐up (hazard ratio [HR]: 2.22, 95% confidence interval [CI]: 1.33−3.70, log‐rank *p* = 0.002). Overall, 237 (38.5%) patients with vascular dementia and 193 (31.4%) patients with AD, developed an ischemic stroke/TIA. The risk of ischemic stroke/TIA was 1.32‐fold higher in vascular dementia (HR: 1.32, 95% CI: 1.09−1.59, log‐rank *p* = 0.003). The risk of rehospitalization (HR: 1.14, 95% CI: 1.01−1.31), and mortality (HR: 1.25, 95% CI: 1.01−1.58) were also higher among AF patients with vascular dementia compared to AD.

**Conclusions:**

The two forms of dementia in AF patients are associated with different prognosis, with AD being associated with a higher risk of ICH, and vascular dementia with a higher risk of stroke/TIA, hospitalization, and mortality.

## Graphical abstract

AF means atrial fibrillation.

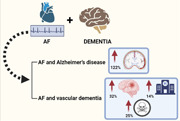

## 1 INTRODUCTION

An increased risk of dementia has been well recognized in patients with atrial fibrillation (AF).^1^, ^2^ Although AF has been predominantly associated with vascular dementia, based on common traditional cardiovascular risk factors, several studies have demonstrated that the risk of dementia persists even in the absence of history of an overt clinical stroke.^1^, ^2^, ^3^ Therefore, it has been hypothesized that multiple mechanisms act in a synergistic manner, including silent cerebral ischemia, hypoperfusion, systemic inflammation, cerebral hypoperfusion, and microbleeds all contributing to the development of cognitive decline in patients with AF.^4^

Recently, brain imaging studies reporting an increased amyloid deposition in patients with AF suggest that not only vascular dementia but also degenerative subtypes of dementia, and more specifically Alzheimer's disease (AD), are associated with AF. More recently a meta‐analysis based on 56 370 patients from six studies reporting on the clinical association between AD and AF, found that patients with AF had a 30% increased chance of developing AD compared with people without AF.^5^ Hence, AF may be linked to both vascular and degenerative dementia, which may also manifest in mixed forms. While the epidemiological association between AF and dementia has been well documented, the clinical implications have been scarcely investigated. Current guidelines on AF recommend the use of oral anticoagulation therapy in patients with dementia according to their CHA_2_DS_2_‐VASc score and good control of risk factors, but do not suggest any clinical utility in differentiating between dementia subtypes.^6^, ^7^

In this study, using a global federated database of electronic health records, our aim was to investigate the association between subtype of dementia (AD or vascular dementia) and the risk of adverse outcomes in patients with AF. We hypothesized that the specific feature of vascular versus AD, as for example amyloid deposition, may confer a different prognosis in AF patients.

## 2 METHODS

The data source used was TriNetX, a global federated health research network with real‐time updates of anonymized electronic medical records (EMRs). The network includes healthcare organizations (HCOs), academic medical centers, specialty physician practices, and community hospitals, with data for >80 million patients predominately based in the United States. To comply with legal frameworks and ethical guidelines guarding against data reidentification, the identity of participating HCOs and their individual contribution to each data set are not disclosed. As a federated research network, studies using the TriNetX health research network do not require ethical approval as no patient identifiable identification is received.

For the present study, the TriNetX research network was searched for the inclusion of AF patients (ICD‐10‐CM code: I48) from 18 to 80 years of age, between January 1, 2000 and 2017. All included patients had AF and a diagnosis of vascular dementia or AD (ICD‐10‐CM codes: F01 and G30, respectively) on or before the diagnosis of AF, to avoid a potential survival bias if AF patients developed de novo dementia. Patients with chronic rheumatic heart disease (ICD‐10‐CM code: I05−I09), acute rheumatic fever (ICD‐10‐CM code: I00−I02), or prosthetic heart valves (ICD‐10‐CM code: Z95.2), were excluded. For comparison of cohorts, patients with AD were excluded from the cohort with vascular dementia and vice‐versa.

Diagnoses were identified using the International Classification of Diseases, Tenth Revision, Clinical Modification (ICD‐10‐CM) codes in the EMRs. The searches were run in TriNetX on April 21, 2022. At the time of the search, there were 67 participating HCOs within the TriNetX research network.

### 2.1 Follow‐up and clinical outcomes

The choice of dates allowed for 4 years follow‐up within the participating HCOs. Outcome measures included incident intracranial hemorrhage (ICH) (ICD‐10‐CM codes: I60, I61, I62), the composite of ischemic stroke/transient ischemic attack (TIA) (ICD‐10‐CM codes: G45, I63, I67.2), hospitalization (ICD‐10‐CM codes: 1013659, 1013699, 1013729), and all‐cause mortality.

### 2.2 Statistical analysis

Continuous variables were expressed as mean and standard deviation (SD), and tested for differences with independent‐sample *t*‐tests. Categorical variables were expressed as absolute frequencies and percentages, and tested for differences using *χ* ^2^ test.

The TriNetX platform was used to run 1:1 propensity score matching (PSM) using logistic regression. The platform uses “greedy nearest‐neighbor matching” with a caliper of 0.1 pooled SDs and difference between propensity scores ≤0.1. Covariate balance between groups was assessed using standardized mean differences (SMDs). Any baseline characteristic with a SMD between cohorts <0.1 is considered well matched.^8^

Hazard ratios (HR) and 95% confidence intervals (CI) were calculated following PSM, and Kaplan−Meier survival curves with log‐rank tests. No imputations were made for missing data. Two‐sided *p* < 0.05 were accepted as statistically significant. Statistical analyses were performed using the TriNetX analytics function in the online research platform.

## 3 RESULTS

### 3.1 Participant characteristics

The search identified 2377 patients with AF and a diagnosis of dementia, which included 1225 patients with vascular dementia (mean age: 66 ± 6.4; 37.3% female) and 1152 with AD (mean age: 67.9 ± 6.1; 43.7% female).

Table 1 summarizes the baseline characteristics of patients with AF and vascular or AD, before and after PSM. Patients with AF and AD were significantly older (66 ± 6.4 vs. 67.9 ± 6.1, *p* < 0.001), mostly of non‐Hispanic White ethnicity and presented a lower cardiovascular risk profile characterized by alower prevalence of hypertension, diabetes, ischemic heart disease, and heart failure, among others (*p* < 0.05 for all). Following PSM, 615 patients were included in each of the two cohorts (i.e., 1:1) and these variables were well‐matched and no longer significantly different (Table 1).

**Table 1:** Comparison of clinical characteristics of the study cohort before and after propensity score matching.

	Initial populations						Propensity score matched populations					
	AF and vascular dementia (*N* = 1225)		AF and Alzheimer's disease (*N* = 1152)		*p* Value	SMD	AF and vascular dementia (*N* = 615)		AF and Alzheimer's disease (*N* = 615)		*p* Value	SMD
Age (years), mean (SD)	66.0	6.40	67.92	6.10	<0.001	0.305	67.21	5.53	67.31	6.37	0.800	0.014
Female sex	456	37.32	504	43.79	0.001	0.132	253	41.14	246	40.00	0.684	0.023
Race												
White	744	60.88	846	73.50	<0.001	0.271	421	68.46	417	67.81	0.806	0.013
Black or African American	275	22.50	113	9.82	<0.001	0.350	99	16.10	77	12.52	0.073	0.102
Comorbidities												
Hypertension	1088	89.03	885	76.89	<0.001	0.327	510	82.93	513	83.42	0.819	0.013
Diabetes mellitus	689	56.38	479	41.62	<0.001	0.298	310	50.41	315	51.22	0.775	0.016
Ischemic heart disease	702	57.45	469	40.75	<0.001	0.338	301	48.94	316	51.38	0.392	0.048
Heart failure	546	44.68	313	27.19	<0.001	0.370	228	37.07	223	36.26	0.767	0.016
Peripheral vascular disease	252	20.62	136	11.82	<0.001	0.240	99	16.10	96	15.61	0.814	0.013
Hyperlipidemia	789	64.57	684	59.43	<0.001	0.106	374	60.81	391	63.58	0.317	0.057
Cerebrovascular disease	944	77.25	356	30.93	<0.001	1.049	343	55.77	346	56.26	0.863	0.009
Arterial embolism and thrombosis	52	4.26	26	2.26	0.006	0.112	21	3.42	22	3.58	0.876	0.008
Pulmonary embolism	75	6.14	50	4.34	0.051	0.080	33	5.37%	34	5.53	0.900	0.007
Other venous embolism and thrombosis	174	14.24	126	10.95	0.015	0.099	73	11.87	73	11.87	1.000	<0.001
Overweight/obesity	322	26.35	235	20.42	<0.001	0.140	149	24.23	146	23.74	0.841	0.011
Diseases of the respiratory system	846	69.23	688	59.77	<0.001	0.198	401	65.20	409	66.50	0.630	0.027
Chronic obstructive pulmonary disease	335	27.41	267	23.20	0.018	0.097	170	27.64	166	26.99	0.797	0.014
Diseases of the digestive system	845	69.15	714	62.03	<0.001	0.150	400	65.04	408	66.34	0.630	0.027
Acute kidney failure and chronic kidney disease	642	52.54	383	33.28	<0.001	0.396	279	45.37	265	43.09	0.421	0.045
Diseases of liver	178	14.57	101	8.78	<0.001	0.181	63	10.24	66	10.73	0.780	0.015
Neoplasms	355	29.05	316	27.45	0.388	0.035	182	29.59	179	29.11	0.850	0.010
Pharmacological therapy												
Beta‐blockers	739	60.48	529	45.96	<0.001	0.294	320	52.03	327	53.17	0.689	0.022
ACE inhibitors	491	40.18	279	24.24	<0.001	0.346	194	31.55	195	31.71	0.951	0.003
Angiotensin II inhibitors	195	15.96	118	10.25	<0.001	0.169	71	11.55	74	12.03	0.790	0.015
Antilipemic agents	667	54.58	444	38.58	<0.001	0.325	281	45.69	288	46.83	0.688	0.022
Calcium channel blockers	510	41.74	329	28.58	<0.001	0.278	202	32.85	207	33.66	0.762	0.017
Diuretics	564	46.15	372	32.32	<0.001	0.286	246	0.04	245	39.84	0.953	0.003
Antiarrhythmics	484	39.61	376	32.67	<0.001	0.144	220	35.77	215	34.96	0.765	0.017
Blood glucose regulation agents (including oral antidiabetics and insulin)	651	53.27	433	37.62	<0.001	0.318	280	45.53	279	45.37	0.954	0.003
Antiplatelets	660	54.01	461	40.05	<0.001	0.282	283	46.02	282	45.85	0.954	0.003
Oral anticoagulants												
Warfarin	377	30.85	252	21.89	<0.001	0.204	158	25.69	153	24.88	0.742	0.018
Apixaban	65	5.32	48	4.17	0.189	0.054	30	4.88	29	4.72	0.893	0.007
Rivaroxaban	66	5.40	60	5.21	0.838	0.008	26	4.23	25	4.07	0.886	0.008
Dabigatran	19	1.56	29	2.52	0.095	0.068	10	1.63	13	2.11	0.527	0.036
Edoxaban	10	0.82	0	0.00	0.002	0.128	0	0.00	0	0.00	NA	NA

Abbreviations: AF, atrial fibrillation; SMD, standardized mean difference.

### 3.2 Risk of incident ICH

**Figure 1:**
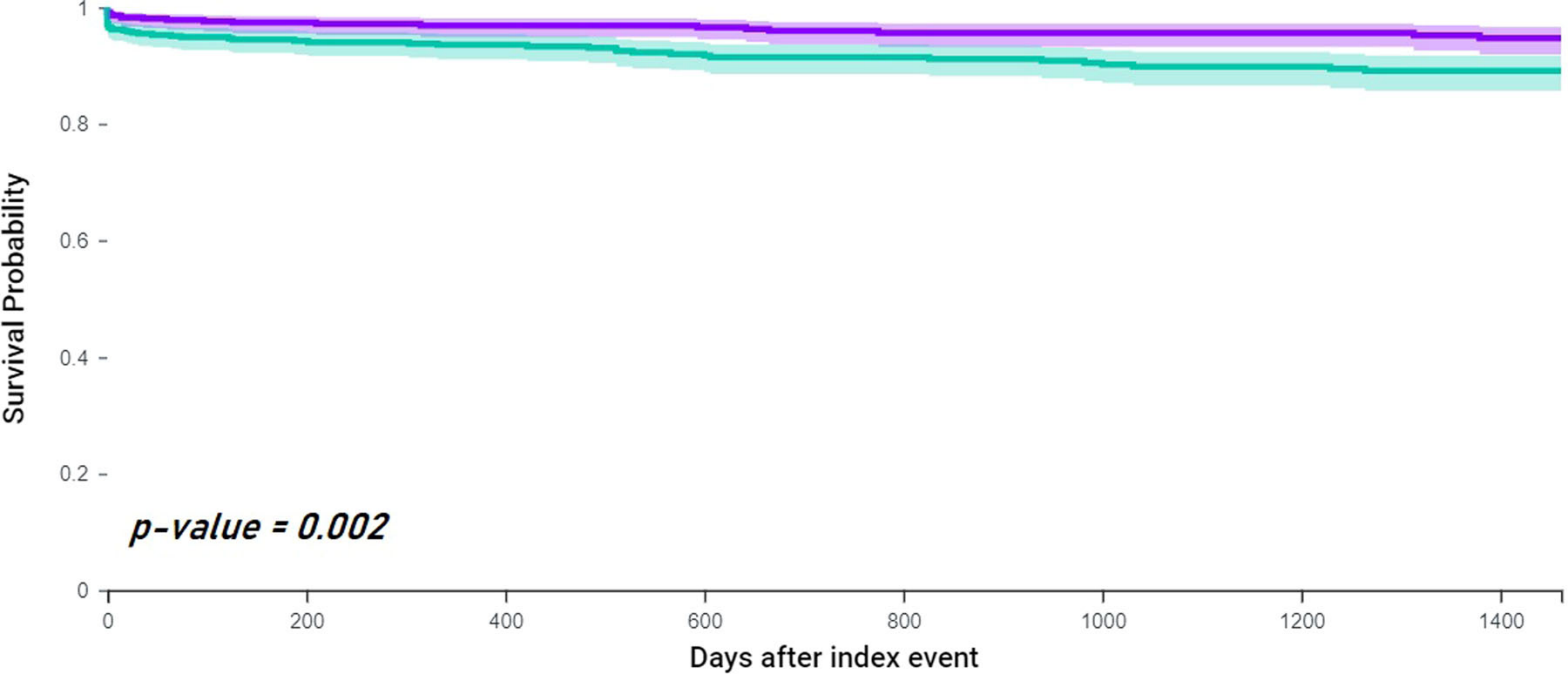
Kaplan−Meyer curves showing survival free from incident intracranial hemorrhage in patients with AF and vascular dementia (green) compared to AF and Alzheimer's disease (purple). AF, atrial fibrillation.

After PSM, 22 (3.6%) AF patients with vascular dementia and 55 (8.13%) patients with AF and AD had incident ICH during the follow‐up. The risk of ICH was higher in patients with AF and AD compared to vascular dementia (HR: 2.22, 95% CI: 1.33−3.70, log‐rank *p* = 0.002) (Figure 1).

### 3.3 Composite incidence of stroke and TIA

**Figure 2:**
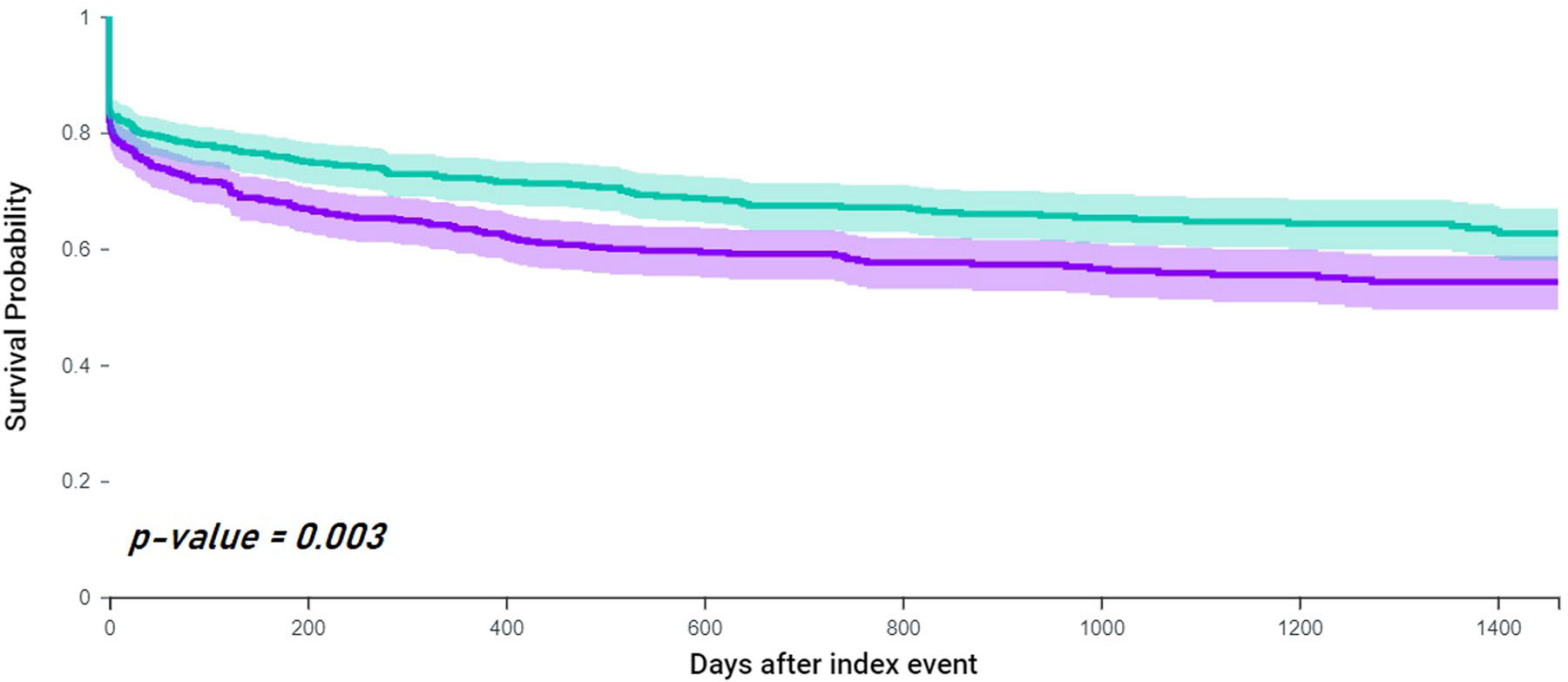
Kaplan−Meyer curves showing survival free from composite of ischemic stroke and transient ischemic attack in patients with AF and vascular dementia (green) compared to AF and Alzheimer's disease (purple). AF, atrial fibrillation.

During the 4‐years of follow‐up, 237 (38.5%) patients with vascular dementia and 193 (31.4%) patients with AD experienced an ischemic stroke/TIA. The risk of ischemic stroke/TIA was 1.32‐fold higher in patients with vascular dementia (HR: 1.32, 95% CI: 1.09−1.59), confirmed in the survival analysis (log‐rank *p* = 0.003) (Figure 2).

### 3.4 Risk of hospitalization

**Figure 3:**
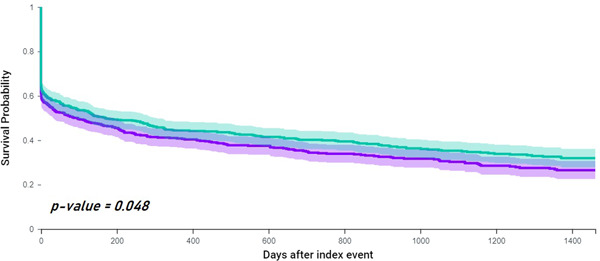
Kaplan−Meyer curves showing survival free from risk of hospitalization in patients with AF and vascular dementia compared to AF (green) and Alzheimer's disease (purple). AF, atrial fibrillation.

During the 4‐years of follow‐up, 406 (66.0%) patients with vascular dementia and 382 (62.1%) patients with AD had hospitalization. The risk of rehospitalization was 1.14‐fold higher in patients with vascular dementia compared to AD (HR: 1.14, 95% CI: 1.01−1.31), confirmed in the survival analysis (log‐rank *p* = 0.048) (Figure 3).

### 3.5 Mortality

**Figure 4:**
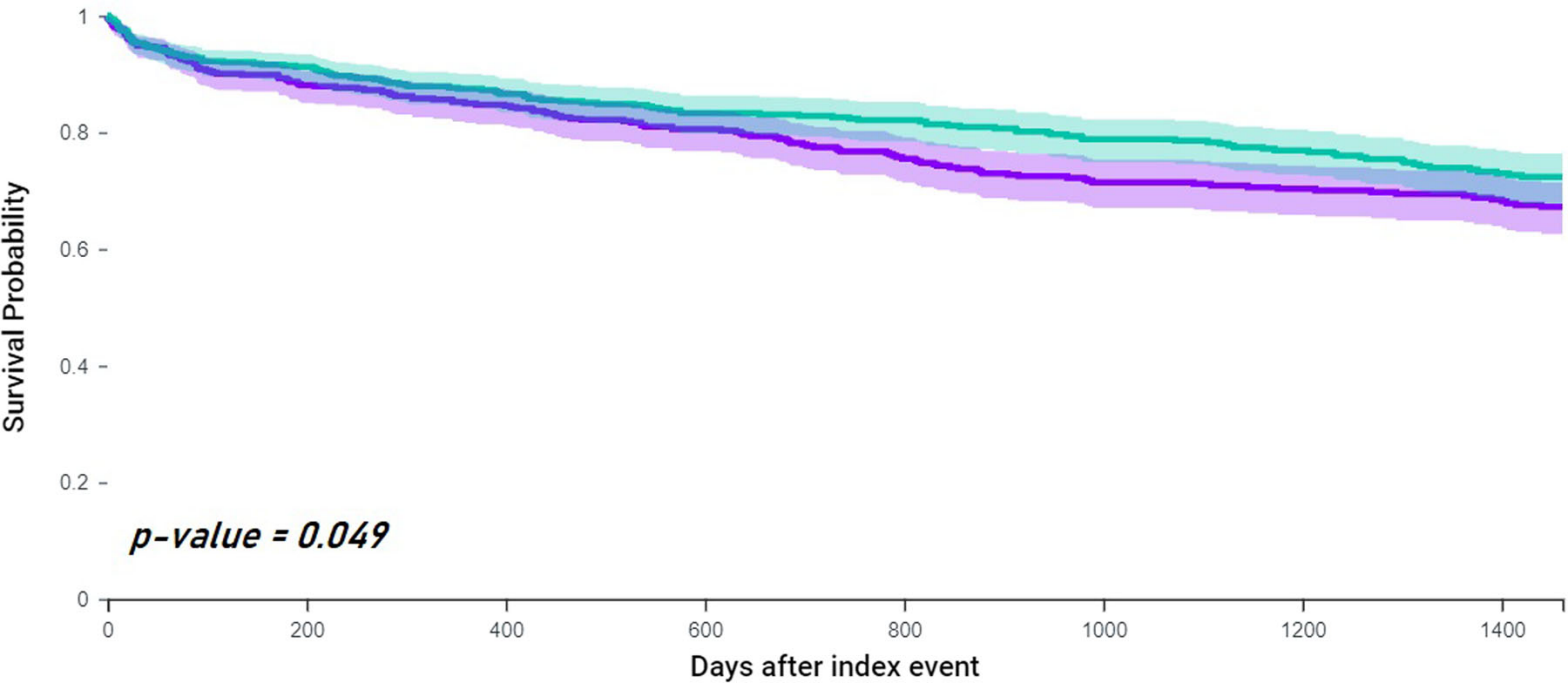
Kaplan−Meyer curves showing survival in patients with AF and vascular dementia (green) compared to AF and Alzheimer's disease (purple). AF, atrial fibrillation.

Mortality was higher among patients with vascular dementia compared to AD (HR: 1.25, 95% CI: 1.01−1.58). Figure 4 reports the survival curves showing that survival was lower in patients with vascular dementia (log rank *p* = 0.049).

## 4 DISCUSSION

In this retrospective analysis of patients with AF from a large global federated data set, there was a similar prevalence of patients with AD and vascular dementia. However, the risk profile and prognosis of these two forms of dementia appear to be different, since patients with vascular dementia presented with a higher risk of ischemic stroke/TIA, rehospitalization, and mortality compared to AD, whereas AD patients had a higher risk of ICH. To the best of our knowledge, there is no previous study comparing the risk of adverse clinical outcomes in patients with AF according to different dementia types.

The differences we detected in our analysis, showing a slightly lower prevalence of AD in African American, are consistent with prior data reporting that missed diagnosis of AD was common in this group and a well documented high prevalence of AD among non‐Hispanic White patients.^9^, ^10^ The higher cardiovascular risk profile of patients with vascular dementia also reflects the higher CHA_2_DS_2_‐VASc risk associated with stroke. It has been already reported that hypertension is not a risk factor for AD^11^; therefore, the significant difference found in the prevalence of hypertension between the two groups is unsurprising. It is worth mentioning that type 2 diabetes has been increasingly recognized as a risk factor for AD through different mechanisms including inflammation, insulin resistance, and hyperglycemia.^9^

Our analysis reports that patients with AF and AD resent a higher risk of non‐traumatic ICH than in those with vascular dementia. However, rate of ischemic stroke/TIA in patients with AD in this cohort (~30%) was not neglectable to justify specific adjustment on anticoagulation therapy in this population because of a higher bleeding risk. Indeed, the increased risk of ICH in patients with AD is aligned with the clinical dilemma of anticoagulation in patients with cerebral amyloid angiopathy given that perivascular amyloid deposition decreased cerebral arterial compliance and cerebrovascular resistance leading to increased susceptibility to micro‐bleeding, hemosiderosis, and ICH.^12^ However, cerebral amyloid angiopathy is a challenging diagnosis mostly linked to magnetic resonance imaging findings. A recent consensus paper did not advise any indication for brain imaging in patients with AF for risk stratification or clinical management.^13^ In contrast, our finding directly correlates the increased risk of ICH with the clinical diagnosis of AD.

On the contrary, the risk of ischemic stroke/TIA was increased in AF patients with vascular dementia. This may be expected because, despite the PSM balancing the comorbidities, patients with vascular dementia may have a higher predisposition to recurrent ischemic and vascular events. The correlation between vascular dementia and stroke has been previously reported though not fully clarified. The risk of dementia in patients who have suffered a stroke is doubled and approximately 30% of stroke survivors will develop cognitive dysfunction within 3 years.^14^ However, many questions linking cerebrovascular disease to dementia remain unanswered including the role of silent cerebral ischemia and cerebral hypoperfusion leading to cortical atrophy. The relationship between cerebrovascular lesions and the susceptibility to amyloid deposition and neural damage that characterize mixed forms of dementia and common in AF requires further investigation.^9^ Cerebrovascular disease has been suggested to contribute to AD neuropathological changes including selective brain atrophy and accumulation of abnormal proteins, such as amyloid‐beta.^12^ The observed higher risk of death and hospitalization in patients with vascular dementia compared to AD may be linked to the predisposition to ischemic events.

Considering our results, there is a need for a more holistic or integrated care approach to patients with AF,^15^ which has been associated with an improved clinical prognosis.^16^ Such an integrated management should also consider dementia risk,^17^ as it not only impacts on cognitive decline and quality of life but also on the risk of other subsequent worse clinical outcomes.

### 4.1 Limitations

Several limitations should be considered when interpreting the results of the current study. First, the participant information is based on EMRs, and from this, a distinction between mixed forms of vascular and degenerative forms could be performed and just vascular and AD was included. In this study, the cohorts were matched for several factors including age, sex, ethnicity, and comorbidities, but residual confounding may still be present and some health conditions may be underreported in EMRs. A further limitation is that although there was a 4‐year follow‐up by examining the EMRs within TriNetX, any events which occurred outside of the TriNetX network may not be well captured.

## 5 CONCLUSION

This retrospective analysis suggests that among patients with AF and dementia, AD was almost as prevalent as vascular dementia. The two forms of dementia in patients with AF are associated with different prognosis, with AD being associated with a higher risk of ICH compared to vascular dementia, and a higher risk of ischemic stroke/TIA, hospitalization, and mortality among those with vascular dementia.

## CONFLICTS OF INTEREST STATEMENT

J. M. R. C.: Consultant for Idorsia Pharmaceuticals LTD. G. Y. H. L.: Consultant and speaker for BMS/Pfizer, Boehringer Ingelheim, and Daiichi‐Sankyo. No fees are received personally. The remaining authors declare no conflict of interest.

## Data Availability

The data that support the findings of this study are not available since derived from the TriNetX research network. These data are not publicly available due to privacy and permission restrictions. Special authorization is required for accessing TriNetX.
